# Third Wave of Influenza A(H7N9) Virus from Poultry, Guangdong Province, China, 2014–2015

**DOI:** 10.3201/eid2109.150635

**Published:** 2015-09

**Authors:** Shumin Xie, Weixin Jia, Yicun Lin, Kaixiang Xing, Xingxing Ren, Wenbao Qi, Ming Liao

**Affiliations:** South China Agricultural University, Guangzhou, China

**Keywords:** influenza virus, viruses, influenza A(H7N9) virus, H7N9, influenza, third wave, reassortants, poultry, Guangdong Province, China

## Abstract

Fourteen influenza A(H7N9) viruses were isolated from poultry or the environment in live poultry markets in Guangdong Province, China during 2014−2015. Phylogenetic analysis showed that all viruses were descended from viruses of the second wave of influenza A(H7N9) virus infections during 2013. These viruses can be divided into 2 branches.

A new influenza A(H7/N9) virus was detected in China on February 19, 2013, and has caused worldwide concern (*1*). Since 2013, the outbreak of this virus in humans has occurred in 3 waves. The third wave began when 2 additional laboratory-confirmed cases of human infection with this virus were detected in Xinjiang Province, China, on September 2, 2014. This wave has continued with increasing numbers of human cases during 2015, including infections in Fujian, Hong Kong, Guizhou, Jiangsu, and Guangdong Provinces. The largest number of human cases has been reported in southern China; >50 infected patients were detected in Guangdong Province January and February (*2*).

The virus has been identified as a novel triple reassortant of avian influenza A(H7N3), A(H7N9), and A(H9N2) viruses and has low pathogenicity in poultry (*3*–*5*). Influenza A(H7N9) virus is now endemic to China, and its continuing reassortment in poultry makes it probable that humans will continue to be infected sporadically.

Because influenza A(H7N9) virus−contaminated live poultry markets (LPMs) are regarded as major sources of human infections with this virus (*6*–*8*), we implemented LPM sampling programs in Guangdong Province and analyzed the evolution of the virus during the third wave. In this study, we also collected samples from chicken farms and integrated epidemiologic and sequence data to infer the genetic diversity and evolution of influenza A(H7N9) viruses found in poultry in Guangdong Province, China.

## The Study

Poultry surveillance for influenza A(H7N9) virus was conducted at LPMs and chicken farms in Guangdong Province (4 LPMs in Guangzhou, 4 LPMs in Dongguan, 1 LPM in Shanwei, 1 LPM in Chaozhou, 2 farms in Huizhou, and 1 farm in Foshan) during September 1, 2014−February 28, 2015. Throat and cloacal swab specimens were collected every 2 weeks. Specific pathogen-free embryonated chicken eggs were used for virus isolation. Hemagglutination-positive isolates, based upon the agglutination of erythrocytes, were collected and were further subtyped by using hemagglutination inhibition assays and reverse transcription PCR.

**Figure 1:**
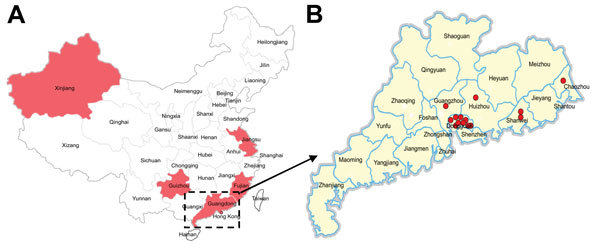
Distribution of influenza A(H7N9) viruses, Guangdong Province, China. A) Shading indicates locations where viruses were isolated from patients during the third wave of the virus mapped according to data from the World Health Organization as of March 1, 2015. B) Circles indicate locations where influenza A(H7N9) viruses were isolated from poultry in Guangdong Province, China, during 2014−2015 (this study).

Fourteen influenza A(H7N9) virus−positive isolates (Figure 1; Technical Appendix Table 1) were sequenced. Full-genome sequences generated in this study were submitted to the **Global Initiative on Sharing All Influenza Data (**GISAID; http://platform.gisaid.org/epi3/frontend#41ab15) under accession nos. EPI_ISL_176816–176820, 176824, 176828, 176830, and 176832–176837.

To understand the molecular epidemiology of these viruses, we compared our data with gene sequences of influenza A(H7N9) viruses in public databases at the National Center for Biotechnology Information (http://www.ncbi.nlm.nih.gov/) and GISAID on March 1, 2015. These data included all available complete gene sequences from influenza A(H7N9) viruses and sequences with high degrees of homology from other subtype viruse gene sequences (hemagglutinin [HA], n = 323; neuraminidase [NA], n = 301; polymerase basic [PB] 2, n = 380; PB1, n = 286; polymerase acidic [PA], n = 286; nonstructural [NS], n = 326; nucleoprotein [NP], n = 311; and matrix [M], n = 316).

Maximum-likelihood trees were estimated for all 8 gene segments by using MEGA version 5.01 (http://www.megasoftware.net). To assess the robustness of individual nodes on phylogenetic trees, a bootstrap resampling process (1,000 replications), the neighbor-joining method, and the maximum composite likelihood model were used.

**Figure 2:**
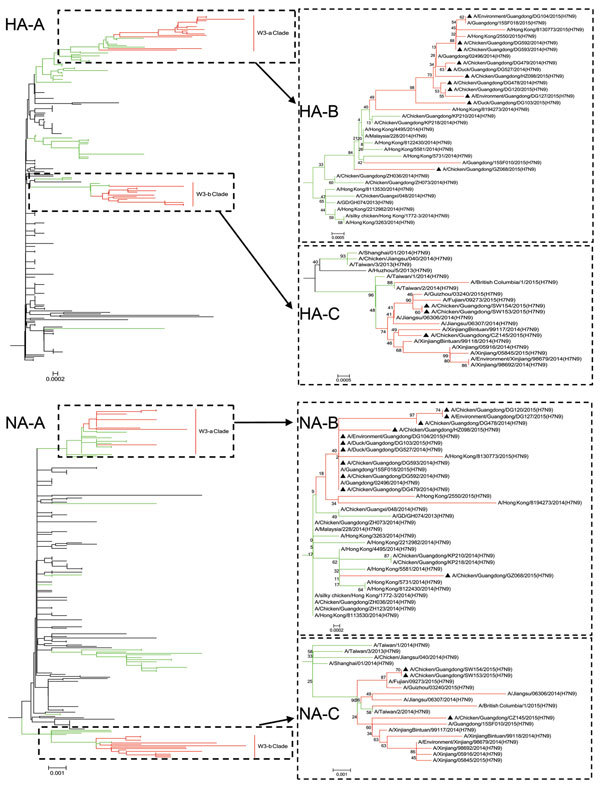
Phylogenetic relationships of influenza A(H7N9) virus hemagglutinin (HA) and neuraminidase (NA) genes isolated from poultry, Guangdong Province, China, 2014−2015. Phylogenetic trees were constructed by using the neighbor-joining method in MEGA software (http://www.megasoftware.net/). B and C are enlargements of A. Branches of the first, second, and third influenza A(H7N9) virus waves are shown in black, green, and red, respectively. Black triangles indicate newly sequenced viruses isolated from poultry in Guangdong during the third wave. Scale bars indicate nucleotide substitutions per site.

Phylogenetic analyses of HA genes confirmed that all third-wave influenza A(H7N9) viruses in Guangdong Province were descended from viruses of the second wave (Figure 2). It is clear that 2 H7N9 lineages co-circulate in Guangdong because third-wave viruses clustered into 2 major clades designated W3-a and W3-b, both of which emerged from the wave 2 clade. The W3-a clade contains viruses detected in Dongguan, Guangzhou, and Huizhou, and clusters of viruses from Guangdong, Hong Kong, and Guangxi, which suggests that W3-a viruses from poultry were simultaneously prevalent in humans residing in these localities. In contrast, A/chicken/Guangdong/GZ068/2015 (H7N9) virus showed major genetic divergence from these viruses.

The W3-b clade contains viruses detected in Shanwei and Chaozhou, including A/chicken/Guangdong/CZ145/2015(H7N9), A/chicken/Guangdong/SW153/2015(H7N9), and A/chicken/Guangdong/SW154/2015(H7N9), that clustered with strains detected in Xinjiang, Fujian, Guizhou, and Jiangsu from humans or the environment during the third wave. These data suggest regional spread of the viruses, probably by regional transport of poultry or by migratory bird populations. Phylogenetic analysis of N9 NA genes showed a topology similar to that of H7 HA genes.

Phylogenetic trees were constructed for each internal gene segment against all currently available H7N9 subtype and other subtype virus sequences (highest homology strains from BLAST [http://blast.ncbi.nlm.nih.gov/Blast.cgi]) from the National Center for Biotechnology Information and GISAID. Phylogenetic analysis of the whole-genome sequences showed that all 6 internal genes of DG478/2014, DG592/2014, DG593/2014, DG479/2014, DG527/2014, HZ098/2015, DG120/2015, and DG127/2015, and the PB1, PB2, PA, and NP genes of DG103/2015, DG104/2015 clustered with strains A/Guangdong/02496/2014(H7N9) and A/Hong Kong/8130773/2015(H7N9) from humans. The NS gene of DG103/2015 clustered with A/Guangdong/15SF018/2015(H7N9). The M gene clustered with A/Hong Kong/8122430/2014(H7N9).

The internal genes of CZ145/2015, SW153/2015, and SW154/2015 showed different genetic characteristics. PB1, PB2, NP, and NS genes of SW153/2015 and SW154/2015 clustered with A/Taiwan/2/2014(H7N9), and M and PA genes were closely related to those of strains isolated in eastern China during the second wave. Internal genes, except for the PA gene of CZ145/2015, clustered with strains isolated from humans in Xinjiang. The PA gene also has a close genetic relationship with the PA gene of an H9N2 subtype strain (A/chicken/Suzhou/097–2/2013).

We conjecture that DG103/2015, CZ145/2015, SW153/2015 SW154/2015, and GZ068/2015 viruses might have undergone additional reassortment, but we cannot infer from our dataset the time, place, or with which other strains these isolates reassorted. Phylogenetic analysis of internal genes also suggested that evolution of wave 3 influenza A(H7N9) viruses resulted in a major increase in genetic diversity and sequential reassortment events with local H9N2 subtype or other subtype viruses (Technical Appendix Figures 1−6).

We conducted mutation analyses of critical and apparent amino acid residues of influenza A(H7N9) virus isolates. All H7N9 subtype viruses isolated have an amino acid PB2-627E, PB2-701D, HA-226L(H3 numbering), NA-289R (N9 numbering), M2-31N, and HA-cleavage sites-PEIPKGRG (Technical Appendix, Table 2). These amino acid residues showed no changes when compared with those of other virus isolates from poultry. All viruses have M2-31N, which might be involved in resistance to adamantane (*9*). Four H7N9 subtype viruses have HA-186V (H3 numbering) and other viruses have HA-186A (H3 numbering). HA-186V may increase binding affinity for the α (2–6)-linked sialic acid receptor (*10*,*11*). PB2-627K can enhance viral replication and virulence in a mice model (*12*), but all H7N9 subtype viruses in our study have PB2-627E. Thus, these strains might be less able to replicate and cause disease in mammals. Although most of the phenotypes associated with the amino acid substitutions have been demonstrated for subtypes other than H7N9, we cannot be sure that these phenotypes are also present in H7N9 subtype viruses.

## Conclusions

Fourteen influenza A(H7N9) viruses were isolated from poultry or environment in LPMs in Guangdong Province, China, during 2014−2015. Phylogenetic analyses of HA and NA genes confirmed that all third-wave influenza A(H7N9) viruses in Guangdong Province were descended from viruses of the second wave. Two H7N9 lineages from poultry co-circulated in Guangdong Province during the third wave, and both are closely related to H7N9 strains isolated from humans in local or adjacent regions. These data suggest that the dominant H7N9 strains have a dynamic evolutionary process for adapting to the local environment. Their internal genes show more regional characteristics, which might be related to transportation of live birds across provinces or to migratory birds.

The results of our study are limited by the number of samples obtained and locations of sampling. However, our findings serve as a warning to public health officials to be aware of the risk of poultry farms being infected with influenza A(H7N9) virus.

**Technical Appendix.** Additional information on influenza A(H7N9) viruses and their internal genes, Guangdong Province, China, 2014−2015.
