# Supplementary material for: Third Wave of Influenza A(H7N9) Virus from Poultry, Guangdong Province, China, 2014–2015
Source: Emerg Infect Dis. 2015 Sep;21(9):1657–60. doi: 10.3201/eid2109.150635 (PMC4550157; doi:10.3201/eid2109.150635)
Supplement: Supplementary file 1 — Technical Appendix. Additional information on influenza A(H7N9) viruses and their internal genes, Guangdong Province, China, 2014−2015. [file 15-0635-Techapp-s1.pdf]

Article DOI: http://dx.doi.org/10.3201/eid2109.150635

# Third Wave of Influenza A(H7N9) Virus from Poultry, Guangdong Province, China, 2014–2015

## Technical Appendix

### Additional Information on Influenza A(H7N9) Viruses and their Internal Genes, Guangdong Province, China, 2014–2015

**Technical Appendix Table 1.** Influenza A(H7N9) viruses isolated and sequenced in this study, Guangdong Province, China*

| ID | Virus | Site | Collection date | Host |
|---|---|---|---|---|
| EPI_ISL_176816 | A/Chicken/Guangdong/DG478/2014(H7N9) | LPM, Houjie, Dongguan | 2014 Nov 25 | Chicken |
| EPI_ISL_176817 | A/Chicken/Guangdong/DG479/2014(H7N9) | LPM, Guancheng, Dongguan | 2014 Nov 30 | Chicken |
| EPI_ISL_176818 | A/Duck/Guangdong/DG527/2014(H7N9) | LPM, Houjie, Dongguan | 2014 Dec 2 | Duck |
| EPI_ISL_176819 | A/Chicken/Guangdong/DG592/2014(H7N9) | LPM, Houjie, Dongguan | 2014 Dec 8 | Chicken |
| EPI_ISL_176820 | A/Chicken/Guangdong/DG593/2014(H7N9) | LPM, Guancheng, Dongguan | 2014 Dec 12 | Chicken |
| EPI_ISL_176824 | A/Chicken/Guangdong/HZ098/2015(H7N9) | Farm, Boluo, Huizhou | 2015 Jan 1 | Chicken |
| EPI_ISL_176828 | A/Duck/Guangdong/DG103/2015(H7N9) | LPM, Humen, Dongguan | 2015 Jan 7 | Duck |
| EPI_ISL_176830 | A/Environment/Guangdong/DG104/2015(H7N9) | LPM, Houjie, Dongguan | 2015 Jan 16 | Environment |
| EPI_ISL_176832 | A/Chicken/Guangdong/DG120/2015(H7N9) | LPM, Guancheng, Dongguan | 2015 Jan 15 | Chicken |
| EPI_ISL_176833 | A/Environment/Guangdong/DG127/2015(H7N9) | LPM, Humen, Dongguan | 2015 Feb 20 | Environment |
| EPI_ISL_176834 | A/Chicken/Guangdong/GZ068/2015(H7N9) | LPM, Jianggao, Guangzhou | 2015 Jan 1 | Chicken |
| EPI_ISL_176835 | A/Chicken/Guangdong/CZ145/2015(H7N9) | LPM, Chaozhou | 2015 Feb 1 | Chicken |
| EPI_ISL_176836 | A/Chicken/Guangdong/SW153/2015(H7N9) | LPM, Shanwei | 2015 Feb 5 | Chicken |
| EPI_ISL_176837 | A/Chicken/Guangdong/SW154/2015(H7N9) | LPM, Shanwei | 2015 Feb 10 | Chicken |

*ID, identification; LPM, live poultry market.

**Technical Appendix Table 2.** Mutation analysis of critical and apparent amino acid residues of influenza A(H7N9) virus isolates, Guangdong Province, China*

| | HA | | | NA | PB2 | | M2 |
|---|---|---|---|---|---|---|---|
| | 186 | 226 | | 289 | | | |
| Virus | (H3 numbering) | (H3 numbering) | Cleavage sites | (N9 numbering) | 627 | 701 | 31 |
| A/Chicken/Guangdong/DG478/2014(H7N9) | A | L | PEIPKGRG | R | E | D | N |
| A/Chicken/Guangdong/DG479/2014(H7N9) | A | L | PEIPKGRG | R | E | D | N |
| A/Duck/Guangdong/DG527/2014(H7N9) | A | L | PEIPKGRG | R | E | D | N |
| A/Chicken/Guangdong/DG592/2014(H7N9) | A | L | PEIPKGRG | R | E | D | N |
| A/Chicken/Guangdong/DG593/2014(H7N9) | A | L | PEIPKGRG | R | E | D | N |
| A/Chicken/Guangdong/HZ098/2015(H7N9) | A | L | PEIPKGRG | R | E | D | N |
| A/Duck/Guangdong/DG103/2015(H7N9) | A | L | PEIPKGRG | R | E | D | N |
| A/Environment/Guangdong/DG104/2015(H7N9) | A | L | PEIPKGRG | R | E | D | N |
| A/Chicken/Guangdong/DG120/2015(H7N9) | A | L | PEIPKGRG | R | E | D | N |
| A/Environment/Guangdong/DG127/2015(H7N9) | A | L | PEIPKGRG | R | E | D | N |
| A/Chicken/Guangdong/GZ068/2015(H7N9) | V | L | PEIPKGRG | R | E | D | N |
| A/Chicken/Guangdong/CZ145/2015(H7N9) | V | L | PEIPKGRG | R | E | D | N |
| A/Chicken/Guangdong/SW153/2015(H7N9) | V | L | PEIPKGRG | R | E | D | N |
| A/Chicken/Guangdong/SW154/2015(H7N9) | V | L | PEIPKGRG | R | E | D | N |

*HA, hemagglutinin; NA, neuraminidase; PB2, polymerase basic 2; M2, matrix 2. HA 186: G→V increases binding affinity for $\alpha$(2–6)–linked sialic acid receptor (*1,2*). HA 226: Q→L increases binding affinity for $\alpha$(2–6)–linked sialic acid receptor (*3–5*). NA 289: R→K reduces susceptibility to oseltamivir (*6*). PB2 627: E→K enhances viral replication and virulence in mice model (*7*). PB2 701: D→N causes mammalian adaptation (*8*). M2 31: S→N causes resistance to adamantanes (*9*).

## References


1. Gambaryan AS, Matrosovich TY, Philipp J, Munster VJ, Fouchier RA, Cattoli G, et al. Receptor-binding profiles of H7 subtype influenza viruses in different host species. J Virol. 2012;86:4370–9. PubMed http://dx.doi.org/10.1128/JVI.06959-11

2. Xiong X, Martin SR, Haire LF, Wharton SA, Daniels RS, Bennett MS, et al. Receptor binding by an H7N9 influenza virus from humans. Nature. 2013;499:496–9. PubMed http://dx.doi.org/10.1038/nature12372

3. Connor RJ, Kawaoka Y, Webster RG, Paulson JC. Receptor specificity in human, avian, and equine H2 and H3 influenza virus isolates. Virology. 1994;205:17–23. PubMed http://dx.doi.org/10.1006/viro.1994.1615

4. Ha Y, Stevens DJ, Skehel JJ, Wiley DC. X-ray structures of H5 avian and H9 swine influenza virus hemagglutinins bound to avian and human receptor analogs. Proc Natl Acad Sci U S A. 2001;98:11181–6. PubMed http://dx.doi.org/10.1073/pnas.201401198

5. Herfst S, Schrauwen EJ, Linster M, Chutinimitkul S, de Wit E, Munster VJ, et al. Airborne transmission of influenza A/H5N1 virus between ferrets. Science. 2012;336:1534–41. PubMed http://dx.doi.org/10.1126/science.1213362

6. Colman PM, Varghese JN, Laver WG. Structure of the catalytic and antigenic sites in influenza virus neuraminidase. Nature. 1983;303:41–4. PubMed http://dx.doi.org/10.1038/303041a0

7. Hatta M, Gao P, Halfmann P, Kawaoka Y. Molecular basis for high virulence of Hong Kong H5N1 influenza A viruses. Science. 2001;293:1840–2. PubMed http://dx.doi.org/10.1126/science.1062882

8. Gao Y, Zhang Y, Shinya K, Deng G, Jiang Y, Li Z, et al. Identification of amino acids in HA and PB2 critical for the transmission of H5N1 avian influenza viruses in a mammalian host. PLoS Pathog. 2009;5:e1000709. PubMed http://dx.doi.org/10.1371/journal.ppat.1000709

9. Pinto LH, Holsinger LJ, Lamb RA. Influenza virus M2 protein has ion channel activity. Cell. 1992;69:517–28. PubMed http://dx.doi.org/10.1016/0092-8674(92)90452-I

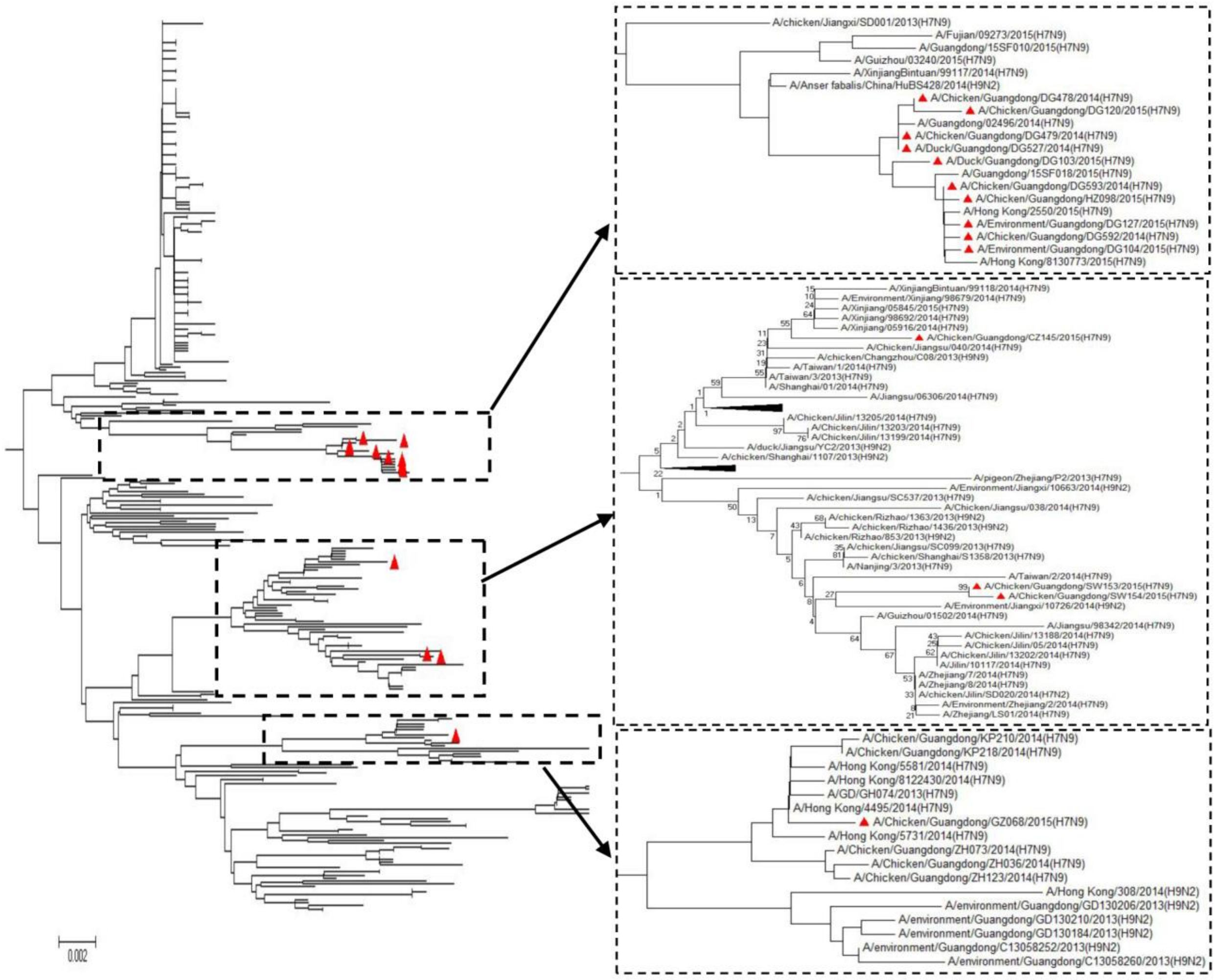


**Technical Appendix Figure 1.** Phylogenetic relationships of polymerase basic 2 genes of influenza A(H7N9) viruses from poultry, Guangdong Province, China, 2014–2015. Trees were constructed by using the neighbor-joining method in MEGA (http://www.megasoftware.net/). Red triangles indicate newly sequenced viruses isolated from poultry in Guangdong during the third wave. Scale bar indicates nucleotide substitutions per site.

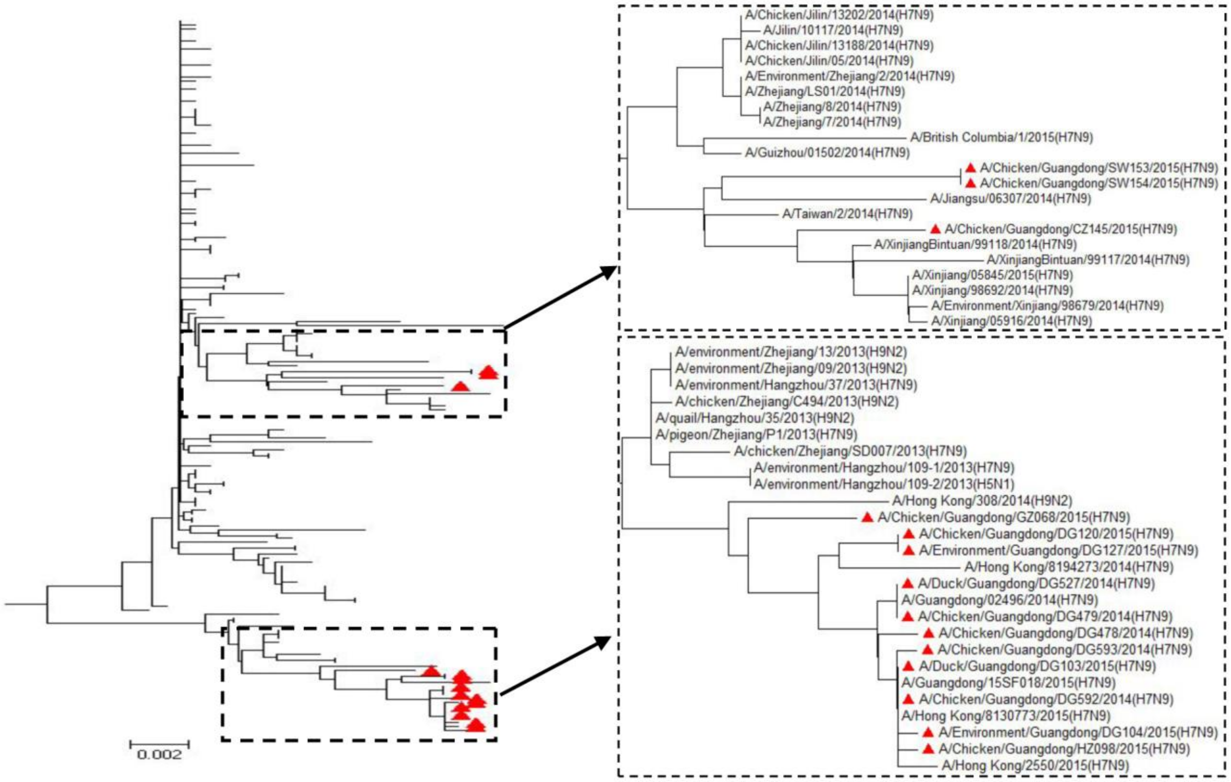


**Technical Appendix Figure 2.** Phylogenetic relationships of polymerase basic 1 genes of influenza A(H7N9) viruses from poultry, Guangdong Province, China, 2014–2015. Trees were constructed by using the neighbor-joining method in MEGA (http://www.megasoftware.net/). Red triangles indicate newly sequenced viruses isolated from poultry in Guangdong during the third wave. Scale bar indicates nucleotide substitutions per site.

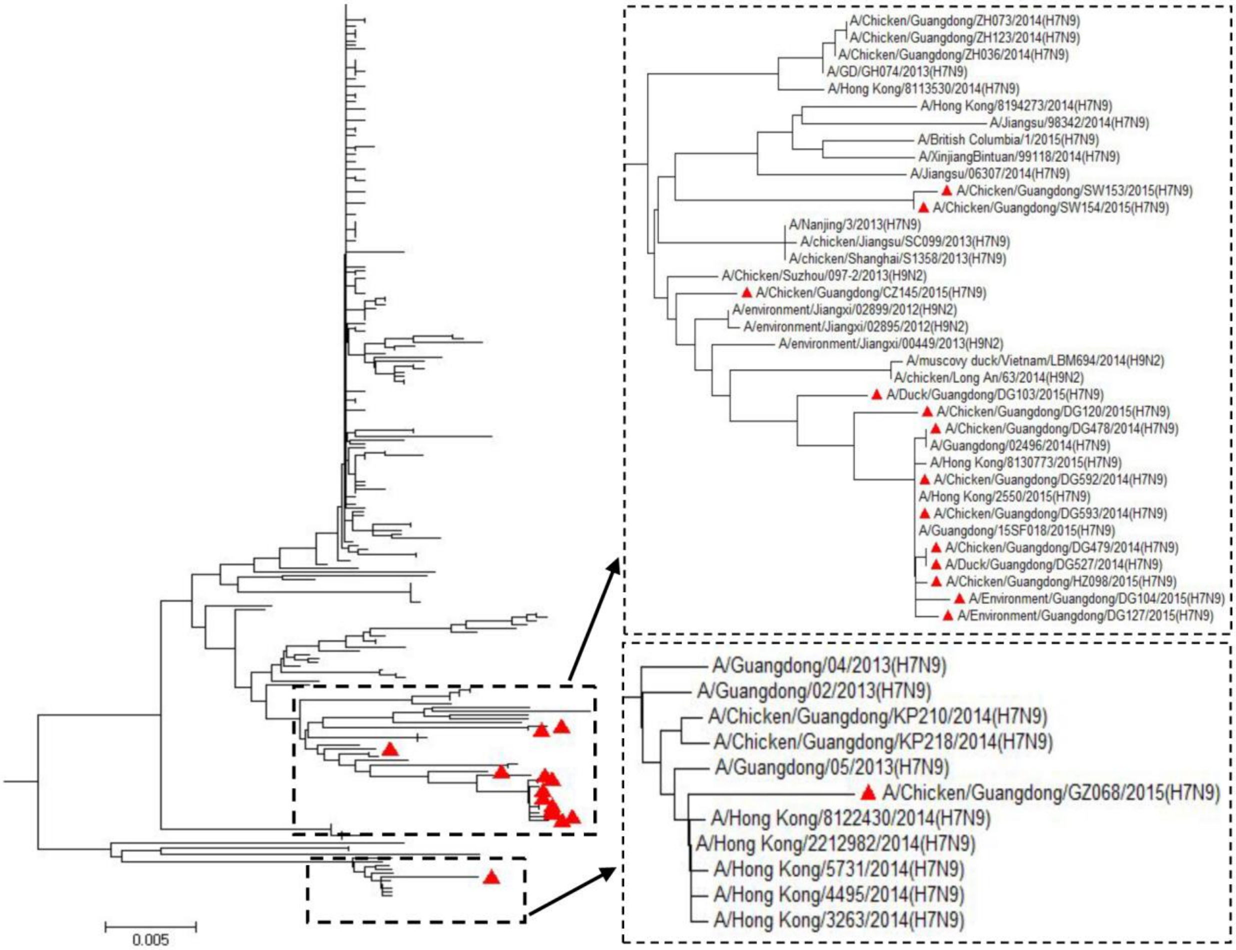


**Technical Appendix Figure 3.** Phylogenetic relationships of polymerase acidic genes of influenza A(H7N9) viruses from poultry, Guangdong Province, China, 2014–2015. Trees were constructed by using the neighbor-joining method in MEGA (http://www.megasoftware.net/). Red triangles indicate newly sequenced viruses isolated from poultry in Guangdong during the third wave. Scale bar indicates nucleotide substitutions per site.

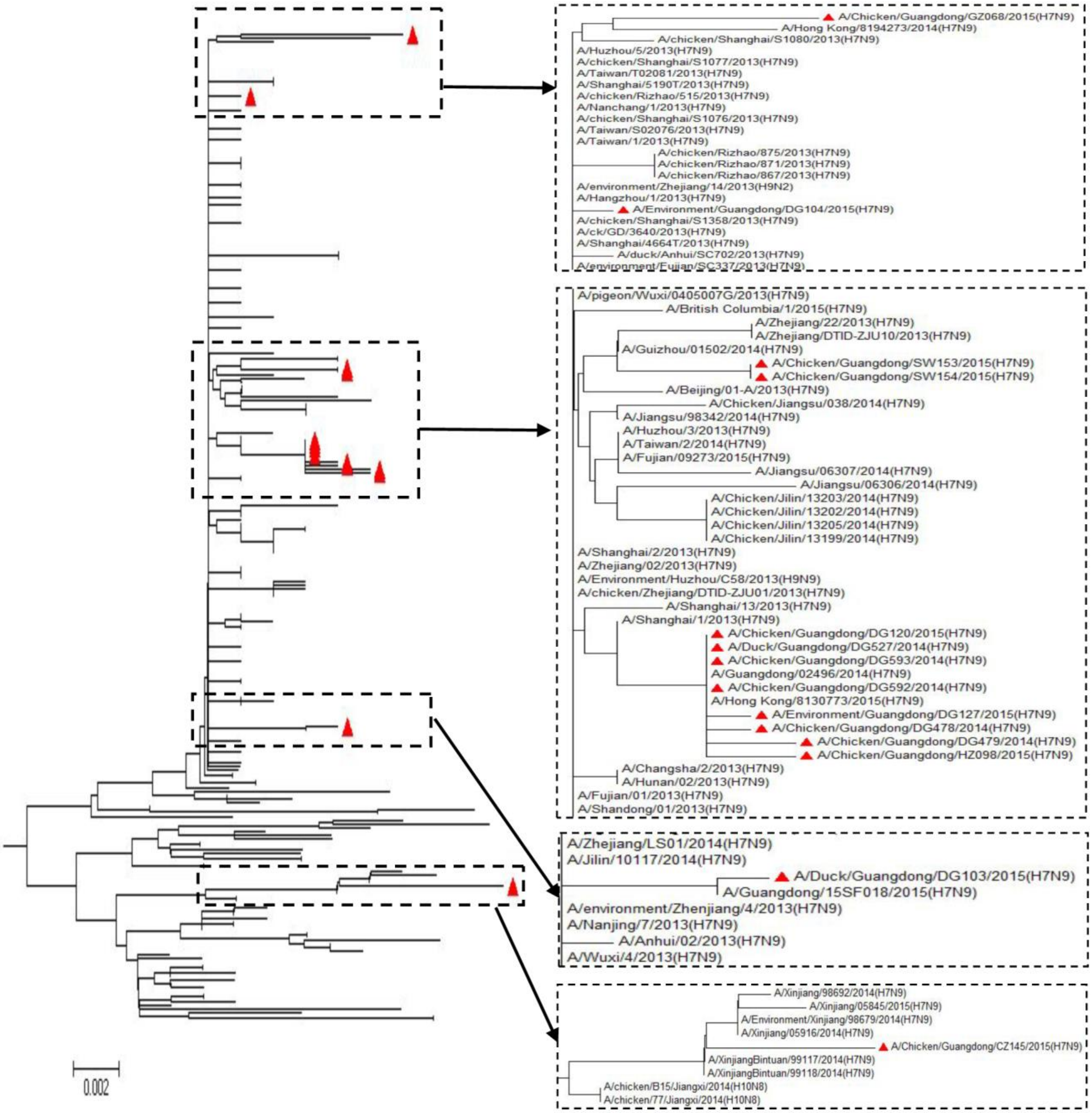


**Technical Appendix Figure 4.** Phylogenetic relationships of nonstructural protein genes of influenza A(H7N9) viruses from poultry, Guangdong Province, China, 2014–2015. Trees were constructed by using the neighbor-joining method in MEGA (http://www.megasoftware.net/). Red triangles indicate newly sequenced viruses isolated from poultry in Guangdong during the third wave. Scale bar indicates nucleotide substitutions per site.

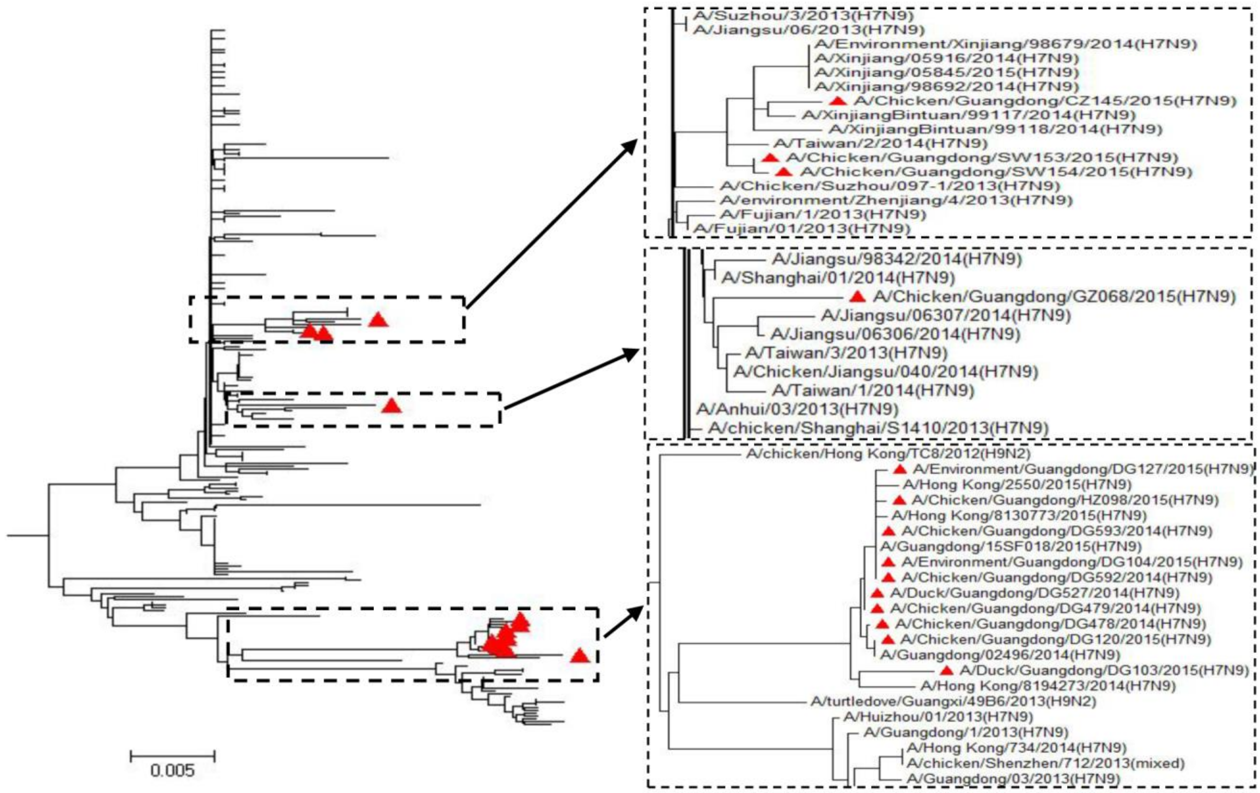


**Technical Appendix Figure 5.** Phylogenetic relationships of nucleoprotein genes of influenza A(H7N9) viruses from poultry, Guangdong Province, China, 2014–2015. Trees were constructed by using the neighbor-joining method in MEGA (http://www.megasoftware.net/). Red triangles indicate newly sequenced viruses isolated from poultry in Guangdong during the third wave. Scale bar indicates nucleotide substitutions per site.

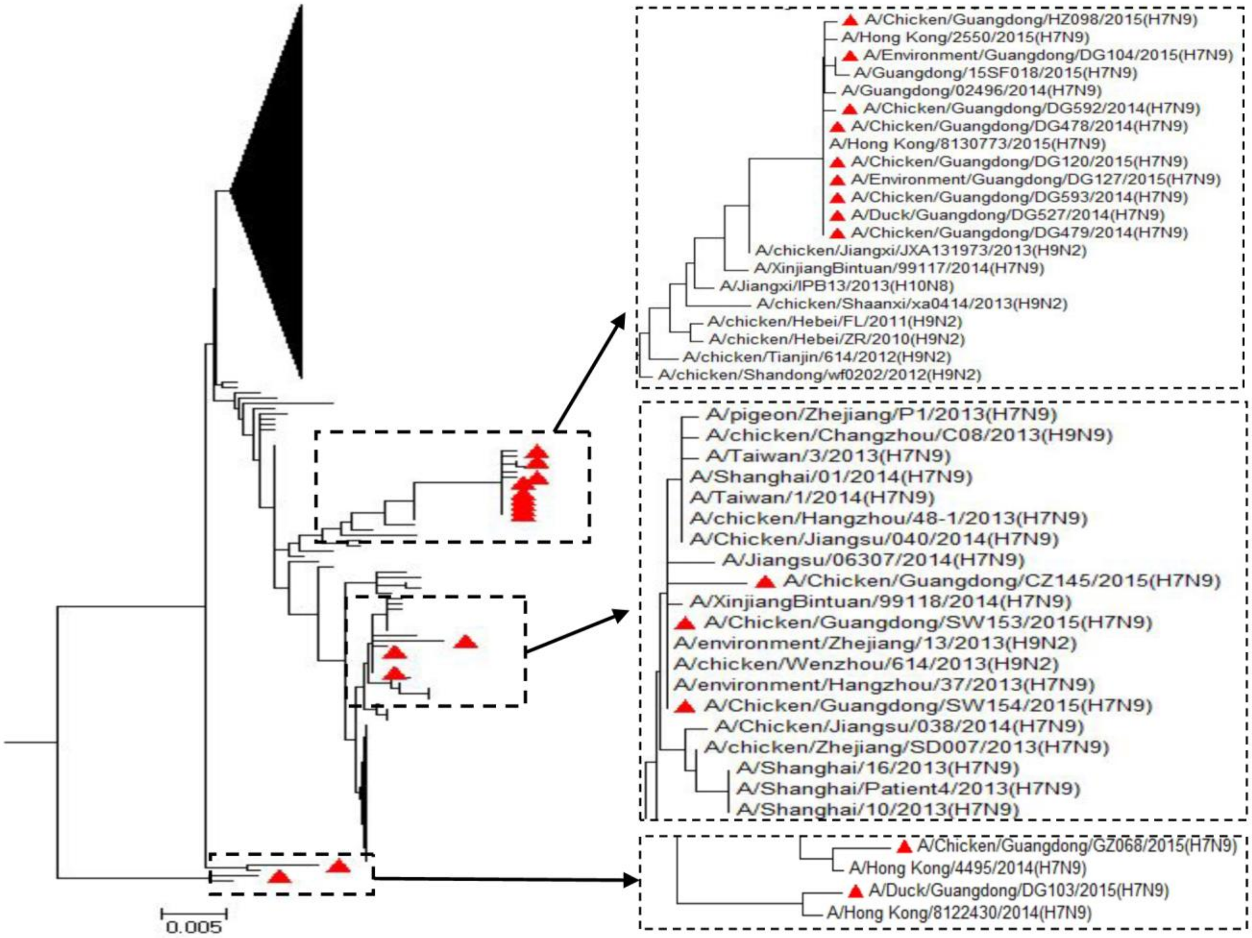


**Technical Appendix Figure 6.** Phylogenetic relationships of matrix genes of influenza A(H7N9) viruses from poultry, Guangdong Province, China, 2014–2015. Trees were constructed by using the neighbor-joining method in MEGA (http://www.megasoftware.net/). Red triangles indicate newly sequenced viruses isolated from poultry in Guangdong during the third wave. Scale bar indicates nucleotide substitutions per site.
